# Ring-shaped calcific constrictive pericarditis strangling the heart: a case report

**DOI:** 10.1186/s12245-014-0040-5

**Published:** 2014-09-30

**Authors:** Mu Sook Lee, Joon Hyouk Choi, Young Uck Kim, Su Wan Kim

**Affiliations:** 1 Department of Diagnostic Radiology, Jeju National University Hospital, Jeju National University School of Medicine, Jeju, 690-767, South Korea; 2 Department of Cardiology, Jeju National University Hospital, Jeju National University School of Medicine, Jeju, 690-767, South Korea; 3 Department of Thoracic and Cardiovascular Surgery, Jeju National University Hospital, Jeju National University School of Medicine, Jeju, 690-767, South Korea

**Keywords:** Constrictive pericarditis, Ring-shaped pericardial calcification, Atrioventricular groove

## Abstract

Constrictive pericarditis is caused by fibrosis and calcification of the pericardium, processes that inhibit diastolic filling of the heart. For the diagnosis of constrictive pericarditis, a combined approach is used to evaluate the morphologic pericardial abnormalities in conjunction with assessment of the functional and hemodynamic changes. We report novel findings of chest computed tomography (CT) and chest roentgenogram with respect to a ring-shaped pericardial calcification on atrioventricular groove causing strangulation of the heart in the patient with constrictive pericarditis, which is anatomically rarer than other severe cases of constrictive pericarditis encasing the entire heart.

## 1 Background

Constrictive pericarditis is the result of scarring and caused by loss of normal elasticity of the pericardial sac. The presence of calcification supports the diagnosis, but not all patients with constrictive pericarditis have calcification [1]. In the past, the main cause of constrictive pericarditis was tuberculosis. However, the spectrum of causes of constrictive pericarditis has changed. Nowadays, the most common causes of constrictive pericarditis are previous open heart surgery and mediastinal irradiation [2]. The diagnosis of constrictive pericarditis has been challenging even though multiple diagnostic modalities have been developed. Pericardial calcifications are considered as an important sign of constrictive pericarditis, which can be depicted in computed tomography (CT) most accurately [1],[3]. Here, we report a case of constrictive pericarditis with a CT finding of a ring-shaped pericardial calcification on atrioventricular groove causing strangulation of the heart, which is anatomically rarer than other severe cases of constrictive pericarditis encasing the entire heart.

## 2 Case presentation

A 66-year-old man was presented with right flank pain, palpitation, gradual dyspnea on exertion, and bilateral leg edema. The patient had a history of atrial fibrillation for 15 years with warfarin intake. He had never undergone thoracic surgery or mediastinal irradiation. An abdominal CT demonstrated a spontaneous bleeding in the right psoas muscle causing the right flank pain. We performed an echocardiogram because the other symptoms representing the heart failure could not be explained. The echocardiogram revealed a constrictive physiology of the mitral and tricuspid valve and a right heart failure with cardiogenic liver cirrhosis. The ratio between early diastolic velocity of lateral mitral annulus and that of septal annulus was significantly reduced. The ratio between early diastolic velocity of right lateral tricuspid annulus and that of septal annulus was also reduced.

**Figure 1:**
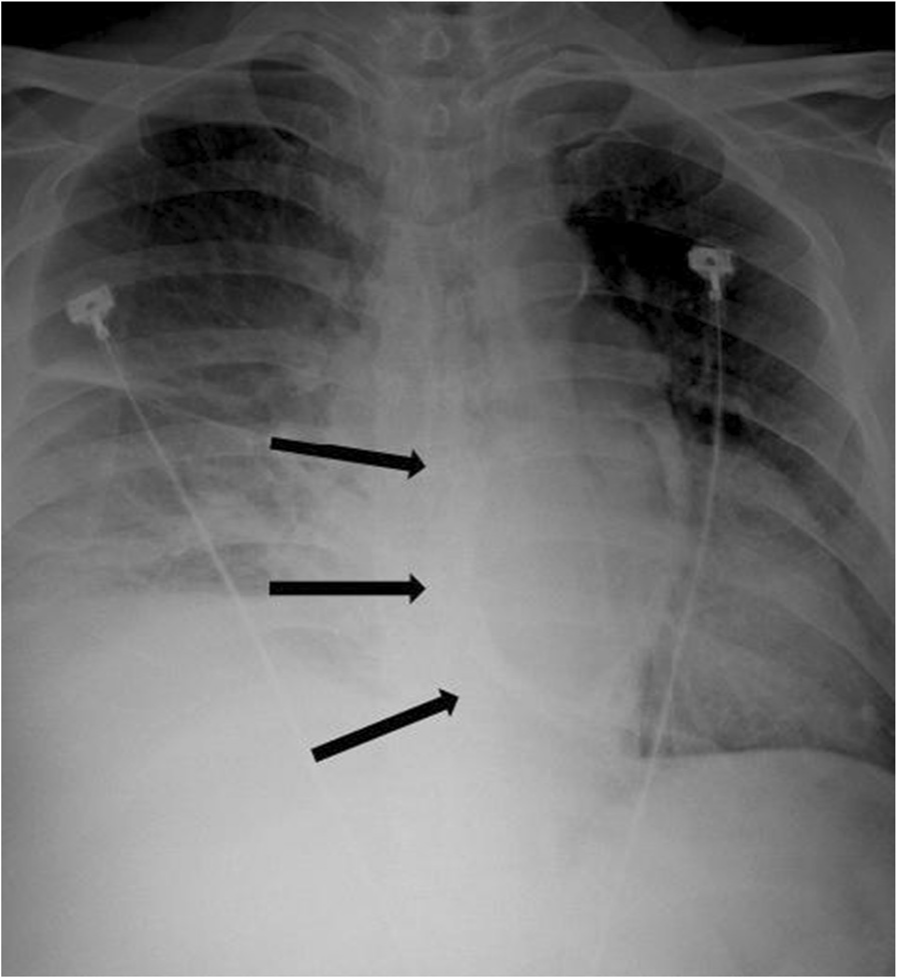
A chest roentgenogram showed a ring-shaped pericardial calcification (arrows).

**Figure 2:**
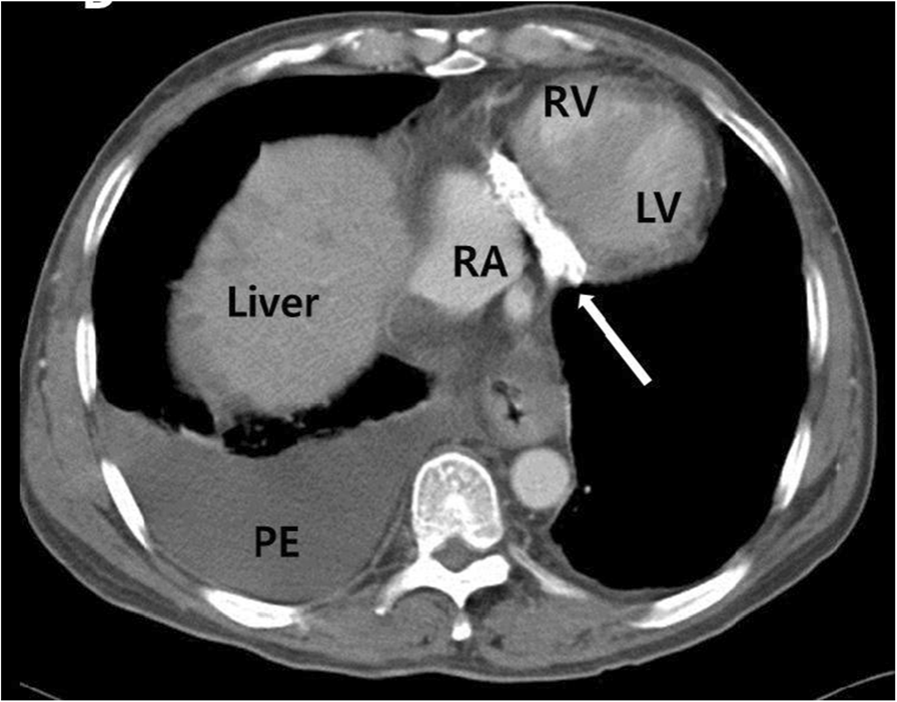
A chest CT revealing dense calcification (arrow) along atrioventricular groove, and pleural effusion (PE).

**Figure 3:**
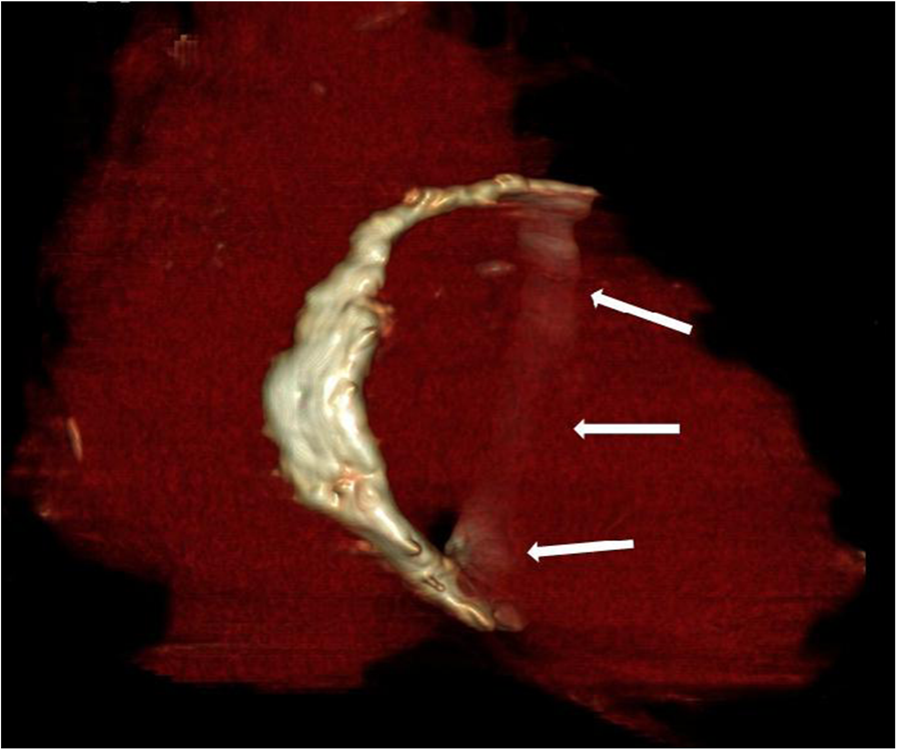
**A reconstructed chest CT demonstrating a ring-shaped massive calcification (arrows) along atrioventricular groove.** It was causing strangulation of the heart

A chest roentgenogram (Figure 1) showed pericardial calcification (arrows). A chest CT (Figure 2) revealed massive calcification (arrows) developed along the atrioventricular groove between the right atrium (RA) and right ventricle (RV) and pleural effusion. A reconstructed chest CT (Figure 3) definitely demonstrated a ring-shaped massive calcification (arrows) along atrioventricular groove causing strangulation of the heart. The patient underwent an extensive pericardial resection through a median sternotomy not using a cardiopulmonary bypass. The calcification of the atrioventricular groove was removed carefully to prevent an injury of the right coronary artery. Histologic study demonstrated calcification and non-specific chronic inflammation with fibrosis. Patient's symptoms of the heart failure improved 3 months after the operation.

## 3 Discussion

Constrictive pericarditis reflects a condition in which the compliance of the pericardium is decreased, which may result in impaired ventricular filling, severe diastolic dysfunction, and right heart failure [1]. The common form of constrictive pericarditis are the subacute elastic constriction and the classic chronic rigid constrictive pericarditis [4]. The spectrum of causes of constrictive pericarditis has changed from tuberculous pericarditis to previous open heart surgery and mediastinal irradiation [2].

The classical presentation of constrictive pericarditis consists of symptoms and signs of debilitating chronic right-side heart failure, such as lower leg edema, hepatic congestion with subsequent development of ascites, anasarca, and jaundice, dyspnea, and orthopnea. However, other cardiac diseases, in particular restrictive cardiomyopathy and tricuspid valve dysfunction can cause signs and symptoms of right-side heart failure similarly [4]. Therefore, a multimodality imaging approach is needed to evaluate the morphologic pericardial and cardiac abnormalities in conjunction with assessment of the functional and hemodynamic consequences [1], even though the most important diagnostic tool is the clinical suspicion of constrictive pericarditis [5].

CT and magnetic resonance (MR) imaging directly demonstrate the typical morphologic manifestation of constrictive pericarditis which is generalized pericardial thickening and pericardial calcifications [1]. A pericardial thickness of >5 to 6 mm is highly specific for constriction and >4 mm is suggestive of pericardial constriction in patients with the appropriate clinical presentation [1]. However, it may be challenging to put pericardial thickening as a necessary criterion for the diagnosis of constrictive pericarditis, because up to 20% of patients with surgically confirmed constrictive pericarditis showed a pericardium of normal thickness on imaging studies [5].

Pericardial calcifications are considered an important finding highly suggestive of constrictive pericarditis on chest radiograph [6]. Ling et al. [7] reported that the presence of pericardial calcification frequently implicates constriction as the cause of the symptoms, regardless of the degree of pericardial thickening. According to Ling et al. [7], 36 of 135 patients with constrictive pericarditis who underwent pericardiectomy demonstrated roentgenological signs of pericardial calcification. In 97% of them, the calcifications were found on the inferior, diaphragmatic surface of the heart, in 76% on the anterior right ventricular area, and in 62% on the left atrioventricular groove. In this case, a chest roentgenogram showed pericardial calcification, and the chest CT confirmed a ring-shaped massive calcification along the whole atrioventricular groove causing strangulation of the heart, which is anatomically rarer than other severe cases of constrictive pericarditis encasing the entire heart [8]. Recently, CT is the most appropriate and accurate tool to depict even minute amounts of calcifications [1]. Our patient presented with signs and symptoms of right-side heart failure and the transthoracic echocardiogram confirmed right-side heart failure with constrictive physiology of the mitral and tricuspid valves. However, it was not easy to assess pericardial thickness on the transthoracic echocardiogram [9]. A multimodality imaging approach including the transthoracic echocardiogram, chest roentgenogram, and CT is helpful for the diagnosis and surgical planning of constrictive pericarditis in our patient.

## 4 Conclusions

Although the diagnosis of constrictive pericarditis is still challenging, a multimodality imaging approach can demonstrate pericardial pathology in detail and help us make a more accurate diagnosis and decide the optimal treatment option for patients with constrictive pericarditis.

This case emphasizes the unique CT findings of ring-shaped calcific constrictive pericarditis and importance of a multimodality imaging approach for the diagnosis of pericarditis.

## 5 Consent

Informed consent was obtained from the patient for publication of this report and companying images.

## Abbreviations

CT: computed tomography

RA: right atrium

RV: right ventricle

LA: left ventricle

PE: pleural effusion

## Competing interests

The authors declare that they have no competing interests.

## Authors’ contributions

ML was involved in preparing and drafting the manuscript and reviewing literature. JC was involved in patient care, providing case details, and revising the manuscript. YK was involved in patient care. SK was involved in patient surgery and revising the manuscript. All authors read and approved the final manuscript.
